# Comparative Analysis of Anterior Segment Parameters in Normal and Keratoconus Eyes Generated by Scheimpflug Tomography

**DOI:** 10.1155/2015/925414

**Published:** 2015-03-24

**Authors:** Faik Orucoglu, Ebru Toker

**Affiliations:** ^1^Birinci Eye Hospital, 34520 Istanbul, Turkey; ^2^Department of Ophthalmology, School of Medicine, Marmara University, 34782 Istanbul, Turkey

## Abstract

*Purpose*. To assess and compare the anterior and posterior corneal surface parameters, keratoconus indices, thickness profile data, and data from enhanced elevation maps of keratoconic and normal corneas with the Pentacam Scheimpflug corneal tomography and to determine the sensitivity and specificity of these parameters in discriminating keratoconus from normal eyes. *Methods*. The study included 656 keratoconus eyes and 515 healthy eyes with a mean age of 30.95 ± 9.25 and 32.90 ± 14.78 years, respectively. Forty parameters obtained from the Pentacam tomography were assessed by the receiver operating characteristic curve analysis for their efficiency. *Results*. Receiver operating characteristic curve analyses showed excellent predictive accuracy (area under the curve, ranging from 0.914 to 0.972) for 21 of the 40 parameters evaluated. Among all parameters indices of vertical asymmetry, keratoconus index, front elevation at thinnest location, back elevation at thinnest location, Ambrósio Relational Thickness (ARTmax), deviation of average pachymetric progression, deviation of ARTmax, and total deviation showed excellent (>90%) sensitivity and specificity in addition to excellent area under the receiver operating characteristic curve (AUROC). *Conclusions*. Parameters derived from the topometric and Belin-Ambrósio enhanced ectasia display maps very effectively discriminate keratoconus from normal corneas with excellent sensitivity and specificity.

## 1. Introduction

Keratoconus (KC) is a noninflammatory ectatic corneal dystrophy characterized by a usually progressive corneal thinning that results in corneal steepening, protrusion, irregular astigmatism, and gradual impairment of vision [1]. Although diagnosis of keratoconus is easy to determine with corneal topography, it is rather difficult to rule out subclinical KC before surgery. Detection of subclinical keratoconus or forme fruste keratoconus among refractive surgery candidates is important because keratorefractive procedures may worsen their condition. Placido disk-based topography systems are sensitive for detecting the subtle changes of topography on the anterior corneal surface. It provides accurate assessment of anterior corneal irregularities in the early stages of keratoconus. Keratoconus indices have been developed to help in detecting and staging keratoconus [2, 3]. Diagnosis and classification criteria for KC are based on anterior corneal curvature data derived with Placido corneal topography. However, it was also reported that early changes in eyes with KC are also present on the posterior corneal surface [4, 5]. Scheimpflug imaging provides the measurement of the entire cornea thickness by determining the front and back surfaces of the cornea taken by a rotating Scheimpflug camera. Keratoconic eyes have thinner corneas than normal eyes, with less volume and a more gradual increase in these parameters from the thinnest point toward the periphery [6]. The combination of the pachymetric graphs and the enhanced elevation maps provided by the Belin-Ambrósio enhanced ectasia display (BAD) of the Scheimpflug system shows sensitivity and specificity in the screening of patients for forme fruste keratoconus eyes [7].

Evaluation of keratoconic and normal eyes to determine all tomographic parameters including keratoconus indices, pachymetric graph values, and back difference elevation values of the corneas may help to identify at-risk corneas.

The purpose of our study was to evaluate and compare the anterior and posterior corneal surface parameters, keratoconus indices, thickness profile data, and data from enhanced elevation maps of keratoconic and normal corneas with the Pentacam Scheimpflug corneal tomography and to determine the sensitivity and specificity of these parameters in discriminating keratoconus from normal eyes.

## 2. Patients and Methods

The protocol of this retrospective clinic-based observational study of 656 eyes of 338 patients diagnosed with keratoconus and 513 eyes of 268 healthy control subjects adhered to the tenets of the Declaration of Helsinki and was approved by the Ethics Committee, Marmara University Hospital, Istanbul, Turkey. All patients included in the study were informed about the purpose of the study and provided informed consent. Subjects were recruited from consecutive patients who were admitted to the university hospital (Marmara University Hospital, Istanbul, Turkey) or the private eye hospital (Birinci Eye Hospital, Istanbul, Turkey) for ocular examination between September 2013 and April 2014.

Exclusion criteria were previous eye trauma, corneal or intraocular surgery, glaucoma, corneal scarring, severe eye dryness, pregnancy or nursing, current corneal infections, and the use of topical medications. Soft contact lens users were included in the study after discontinuation of lens wear for at least 7 days.

Keratoconus was diagnosed mainly on the basis of clinical slit-lamp findings, keratometry, and associated characteristic topographic patterns. Eyes were considered normal if they had no ocular pathology, no previous ocular surgery, and no irregular corneal pattern.

A comprehensive ocular examination including Scheimpflug corneal tomography was performed on all eyes. Measurements were taken with a high-resolution imaging system which uses a rotating Scheimpflug camera (Pentacam, Oculus Optikgeräte GmbH, Wetzlar, Germany) and a monochromatic slit-light source that rotate together around the optical axes of the eye for measuring anterior segment topography. The Pentacam provides a multitude of corneal topographic (keratometric), topometric, tomographic, and pachymetric data. The room lights were switched off for all examinations to get a reflex-free image. The subjects were asked to position themselves, blink a couple of times, and fixate on the black target in the center of the blue fixation beam. Patients were instructed to close their eyes between shots for at least 10 seconds to moisten the eyes. The images were obtained with the automatic mode. The camera was rotated 180°, obtaining 25 slit images of the anterior segment, and generated a three-dimensional model of the anterior eye. Eye movement of the subject was constantly monitored by the system, and quality factor was automatically evaluated. Only the scan results with quality factor (QS) of >95% were saved.

**Figure 1:**
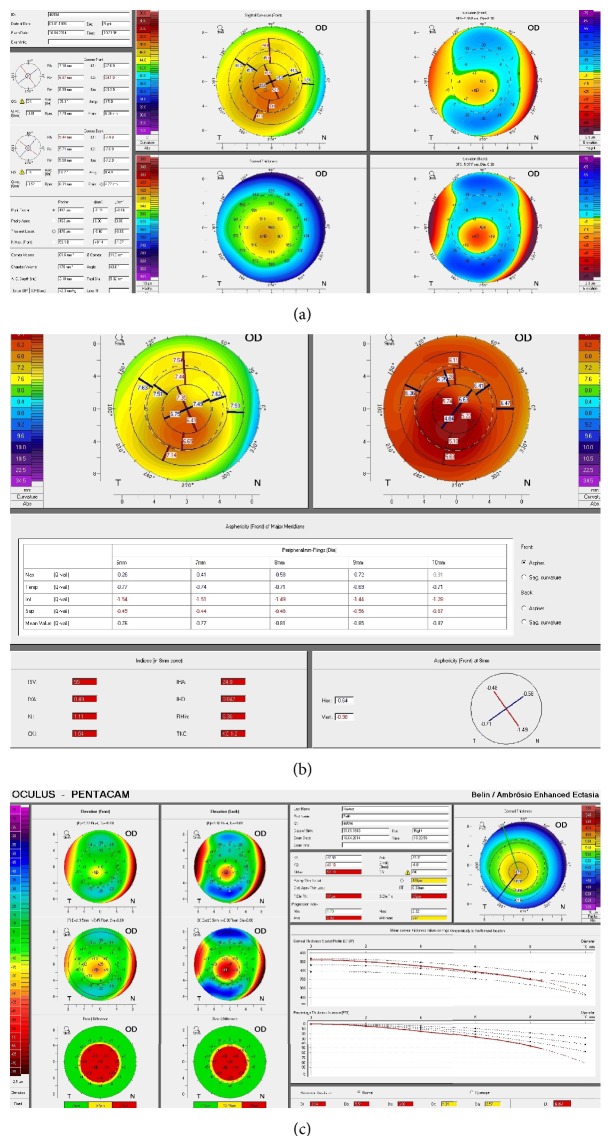
(a) Topographic, (b) topometric, and (c) Belin-Ambrósio enhanced ectasia display maps of the Pentacam.

Parameters were derived from topographic, topometric, and BAD maps (Figures 1(a), 1(b), and 1(c)). Parameters of the printout retained for the analysis were keratometry readings, topographic astigmatism and asphericity for the anterior and posterior corneal surface, pachymetry, cornea volume, and anterior chamber volume, angle, and depth, topometric indices, data from corneal thickness spatial profiles, and Belin-Ambrósio enhanced ectasia display. The abbreviations for these parameters are used in this paper and they are explained in Abbreviations.

Corneal thickness was defined as the thinnest point in the corneal thickness map. Corneal volume is reported as the volume of the cornea in a diameter of 10 mm, centered on the anterior corneal apex. Anterior chamber depth was defined as the distance from the corneal endothelium to the anterior surface of the lens capsule. The anterior chamber volume is calculated from endothelium down to iris and lens over a 12 mm diameter centered on the anterior corneal apex. The default angle displayed is the smallest angle in the horizontal position calculated from the Scheimpflug image. For elevation data measurement, the best fit sphere served as a reference body using the float option and the diameter of the reference surface was 8 mm. Front and back elevation difference values were taken as the differential changes in corneal elevation between the best fit sphere (BFS) and the enhanced BFS obtained with the BAD display software. Progression index is calculated as the average progression value at the different pachymetric rings, referenced to the mean curve.

Spherical equivalent (SE: sphere + half the cylinder) values, in diopters (D), were calculated from cycloplegic refraction for each patient. The asphericity data provided by the Pentacam was taken from 8 mm central cornea with reference to the anterior corneal apex.

Eyes with keratoconus were compared with normal corneas in separate series of analyses. All numerical results were entered into a database, and statistical analysis was performed using Statistical Package for Social Sciences (SPSS) version 16.0. ROC curves were used to determine the overall predictive accuracy of the test as described by the area under the curve. For the output values of the discriminant functions tested, the area under the ROC curve (AUROC), sensitivity (true positive/(true positive false negative)), specificity (true negative/(true negative false positive)), accuracy ((true positive true negative)/total number of cases), and cutoff value were calculated.

The AUROC curve is a plot of sensitivity against 1 − specificity, that is, true positives versus false positives. This area ranges from 1 (100%) representing perfect discrimination to 0.5 (50%) representing discrimination being no better than chance. In between that range, 0.90–1 represent excellent discrimination, 0.80–0.90 good, 0.70–0.80 fair, 0.60–0.70 poor, and 0.50–0.60 very poor [8]. An area of 0.5 represents a completely inefficient measure.

## 3. Results

In the normal group, the study involved 513 eyes of 268 subjects with a mean age of 32.99 years, ranging from 8 to 74 years old. One hundred nine (48.1%) of subjects were male and 139 (51.9%) were female. In the keratoconus group, the study involved 656 eyes of 338 subjects with a mean age of 31.18 years, ranging from 13 to 64 years old. Two hundred fourteen (63.2%) of subjects were male and 124 (36.8%) were female. The mean spherical refraction of normal eyes was −0.88 D.

The two groups did not differ significantly with regard to age and gender (*P* = 0.086 and *P* = 0.09, resp.).

The mean Pentacam parameters and the differences between keratoconus and normal subjects are shown in Table 1.

All parameters derived from the three maps showed statistically significant difference between keratoconic and normal eyes except the distance from corneal apex to thinnest location parameter (*P* = 0.349).

Table 2 shows the results of the ROC curve analysis, standard error, 95% confidence intervals, significance level, best cutoff point, and sensitivity and specificity of best cutoff points for each parameter tested in keratoconus group versus normal eyes.

Out of 40 parameters derived from the topographic, topometric, and BAD maps 2 (TCT, AUROC 0.915; Kmax, AUROC 0.928), 5 (ISV, AUROC 0.954; IVA, AUROC 0.963; KI, AUROC 0.970; IHD, AUROC 0.951; Rmin, AUROC 0.929), and 14 (FDE, AUROC 0.910 BDE, AUROC 0.954; F.Ele.Th, AUROC 0.959; B.Ele.Th, AUROC 0.967; ProgMin, AUROC 0.935; ProgMax, AUROC 0.964; ProgAvg, AUROC 0.955; ARTmac, AUROC 0.961; Df, AUROC 0.949; Db, AUROC 0.957; Dp, AUROC 0.954; Dt, AUROC 0.914; Da, AUROC 0.964; D, AUROC 0.972) parameters, respectively, showed excellent AUROC values in discriminating keratoconic eyes from normal ones.

Among these parameters IVA, KI, F.Ele.Th, B.Ele.Th, ARTmac, Dp, Da, and D showed excellent (>90%) sensitivity and specificity in addition to excellent AUROC. The cutoff points derived from the ROC curve analysis were 0.255 for IVA, 1.055 for KI, 5.5 for F.Ele.Th, 13.5 for B.Ele.Th, 311 for ARTmac, 1.855 for Dp, 1.62 for Da, and 2.615 for D.

## 4. Discussion

This retrospective study analyzed the efficacy of the parameters derived from the anterior and posterior corneal surfaces, keratoconus indices, thickness profile data, and data from enhanced elevation maps in discriminating eyes with KC from normal eyes. All parameters derived from the three maps showed statistically significant difference between keratoconus and normal group except the parameter of distance between thinnest point and apex. Previous studies found a mean distance between the apex and the thinnest point to range from 0.52 to 1.01 mm in healthy eyes and 0.78 mm in keratoconus eyes [9–12]. The mean values in this study in normal (0.82) and KC corneas (0.84) are comparable with previous reports. The differences in the reported values of distance between apex and thinnest location may be due to the high variability of study populations and the variety of instruments used in each study. Rüfer et al. also mentioned that repetition accuracy for the location of the thinnest point was rather poor, based on a high standard deviation of *x* and *y* coordinates, and attributed this to minor fixation deviations of the subject's eyes [13].

We further did ROC analysis to evaluate the predictive accuracy of these parameters in differentiating KC from normal corneas. Forty parameters derived from three maps were analysed. Out of 15 parameters from the topography maps thinnest corneal thickness (TCT) and Kmax showed excellent predictive accuracy. Being a well-known pathophysiological feature of KC, corneal thickness is an important marker for both detection of KC and the severity level of the disease [14, 15]. In this study, a cutoff value of 506 *μ* had 89.0% sensitivity and 83.2% specificity for discriminating normal eyes from keratoconus. In previous studies, the cutoff point of TCT ranged from 489 to 493 *μ* [7, 16, 17].

Nine parameters were studied from the topometric map and 5 of them showed excellent discrimination of keratoconus. These anterior surface topometric indices were ISV, IVA, KI, IHD, and Rmin.  Table 3 shows abnormal topometric indices values of the manufacturer's user manual and this study. According to the manufacturer's user manual, an ISV value higher than 37 is considered abnormal (marked with yellow) and higher than 41 is pathological (marked with red) [18]. The cutoff value for ISV in our study was 31.5 with 87.8% sensitivity and 96.2% specificity. An IVA value higher than 0.28 is considered abnormal and higher than 0.32 is pathological [18]. However, our cutoff value was 0.255 with a sensitivity and specificity of 91.3% and 96.4%, respectively. A KI value higher than 1.07 is considered abnormal and/or pathological and IHD value higher than 0.014 is considered abnormal and higher than 0.016 is pathological while we found a cutoff value 1.055 with 91.0% sensitivity and 98.2% specificity for KI and 0.0175 with 90.0% sensitivity and 89.0% specificity for IHD. The average radius of the anterior corneal surface in normal corneas was reported to be 7.87 ± 0.27 mm and it was considered abnormal and/or pathological if it is less than 6.71 mm [19, 20]. In this study, the average value of *R*_min⁡_ was 7.55 ± 0.27 mm in normal eyes and the cutoff value was 7.085 (96.8% sensitivity and 80.7% specificity).

Fifteen parameters were studied from the BAD and 14 of them showed excellent discrimination. Difference in anterior and posterior elevation (FDE, BDE), F.Ele.Th, B.Ele.Th, ProgMin, ProgMax, ProgAvg, ARTmac, Df, Db, Dp, Dt, Da, and D showed excellent predictive accuracy. The BAD is an integrated display in the Pentacam that combines elevation based and comprehensive pachymetric corneal evaluation in an all-inclusive display. The BAD displays each parameter and individually reports them as a standard deviation and then reports a final overall reading that is based on a regression analysis to maximize the separation of normal corneas from those with keratoconus [21]. Fam and Lim showed that anterior corneal elevation parameters are clinically relevant measures for detecting keratoconus and suspected keratoconus eyes [22]. Previous studies reported that anterior and posterior elevation were the most effective parameters for the diagnosis of keratoconus [23, 24]. In accordance with previous reports, the results of our study showed that anterior and posterior elevation parameters have excellent predictive accuracy in detecting keratoconus. The cutoff value for anterior elevation at the thinnest point in our study was 5.5 (91.3% sensitivity and 97.4% specificity) and it was 13.5 (93.2% sensitivity and 94.9% specificity) for posterior elevation which was comparable to that reported by Muftuoglu et al. but still lower than that reported by de Sanctis et al. [25, 26].

Corneal thickness spatial profile, percentage increase in thickness, percentage increase, and D parameters had high predictive accuracies in discriminating keratoconus from normal eyes.

Ambrósio et al. introduced the analysis of corneal thickness spatial profiles and demonstrated significant differences in absolute thickness and percentage thickness increase as a function of distance from the thinnest point between normal and KC eyes [27, 28]. The ART is a novel combined parameter that combines the thickness and pachymetric distribution and it was reported to have a sensitivity of 100% and a specificity of 96.5% in discriminating keratoconus from normal corneas [28]. The ARTmax provided the best combination of sensitivity (96.6%) and specificity (90.7%) in our study and the cutoff value of 311 was comparable with that reported by Ambrósio et al. [28].

D parameters compute the deviation from normal indices for the enhanced front and back elevations, for the thinnest value and for the pachymetric distributions. We found that all D parameters showed excellent accuracy in the discrimination of keratoconus.

Among all parameters studied, only IVA, KI, F.Ele.Th, B.Ele.Th, ARTmax, Dp, Da, and D showed excellent (>90%) sensitivity and specificity in addition to excellent AUROC level.

One limitation of our study is that forme fruste keratoconus patients were not separated from keratoconus patients, so our work gives no threshold value for the detection of this condition.

In conclusion, this study aimed to evaluate the effectiveness of a large number of Pentacam parameters in the diagnosis of keratoconus in a fairly large sample size showing that the predictive accuracy of topographic, topometric, and BAD parameters was overall high but that the parameters of index of vertical asymmetry, keratoconus index, front and back elevation at the thinnest point, ART, and D values were the most sensitive and specific parameters for the diagnosis of keratoconus. These results may suggest that rather than relying on a single map, comprehensive analysis of topography, topometric indices, pachymetric data, and corneal height data in the Belin-Ambrósio enhanced ectasia display may provide useful information for improving the accuracy of keratoconus diagnosis and screening refractive candidates in a clinical setting.

## Figures and Tables

**Table 1:** Mean Pentacam parameters and the difference between keratoconus and normal eyes.

Pentacam parameter	KC Mean ± SD (range)	Control Mean ± SD (range)	*P*
Topographic map			
Kflat (Ant.)	46.37 ± 4.75 (38.40–72.60)	43.03 ± 1.57 (38.90–47.70)	<0.001
Ksteep (Ant.)	49.41 ± 5.56 (40.30–82.10)	44.17 ± 1.58 (40.10–49.40)	<0.001
Kmean (Ant.)	47.80 ± 5.06 (39.50–77.10)	43.60 ± 1.52 (39.70–48.00)	<0.001
Kmax	54.11 ± 8.34 (42.10–110.50)	44.67 ± 2.17 (12.30–50.20)	<0.001
Astigmatism (Ant.)	3.05 ± 1.97 (0.00–17.00)	1.13 ± 0.86 (0.00–6.10)	<0.001
Asphericity (Ant.)	−0.78 ± 0.54 (−6.90–0.17)	−0.34 ± 0.12 (0.83–0.30)	<0.001
Kflat (Post.)	−6.82 ± 0.96 (−11.90–5.00)	−6.13 ± 0.26 (−7.00–5.30)	<0.001
Ksteep (Post.)	−7.39 ± 1.08 (−13.50–0.50)	−6.41 ± 0.28 (−7.40–5.70)	<0.001
Kmean (Post.)	−7.09 ± 0.95 (−12.50–5.30)	−6.27 ± 0.24 (−7.00–5.50)	<0.001
Astigmatism (Post.)	0.71 ± 0.44 (0.00–4.40)	0.31 ± 0.17 (–0.30–1.00)	<0.001
Asphericity (Post.)	−0.81 ± 0.52 (−3.79–0.89)	0.35 ± 0.15 (−0.90–0.19)	<0.001
TCT	456.76 ± 54.53 (171–626)	545.94 ± 36.76 (443–656)	<0.001
Cornea Vol	57.08 ± 3.71 (47.20–70.60)	60.70 ± 4.06 (49.80–75.60)	<0.001
AC Vol	195.90 ± 35.12 (100–287)	175.04 ± 42.37 (63.00–289)	<0.001
ACD	3.32 ± 0.35 (1.96–4.75)	3.00 ± 0.41 (1.81–4.18)	<0.001
AC Angle	39.20 ± 6.59 (16.90–66.90)	37.01 ± 8.13 (4.86–73.30)	<0.001
Topometric map			
ISV	76.10 ± 42.42 (10–289)	17.70 ± 6.67 (5.00–52.00)	<0.001
IVA	0.77 ± 0.44 (0.02–2.44)	0.12 ± 0.058 (0.01–0.42)	<0.001
KI	1.19 ± 0.13 (1.00–2.11)	1.01 ± 0.019 (0.95–1.07)	<0.001
CKI	1.05 ± 0.057 (0.90–1.37)	1.00 ± 0.005 (0.97–1.02)	<0.001
IHA	22.64 ± 18.96 (0.20–131.90)	4.13 ± 4.60 (0.00–28.60)	<0.001
IHD	0.75 ± 0.070 (0.00–0.67)	0.011 ± 0.019 (0.00–0.24)	<0.001
Rmin	6.35 ± 0.81 (3.05–8.02)	7.55 ± 0.27 (6.72–8.37)	<0.001
HOR Q	−0.85 ± 1.50 (–37–0.28)	−0.33 ± 0.14 (−0.88–0.36)	<0.001
VERT Q	−1.01 ± 3.66 (−69–1.78)	−0.35 ± 0.16 (−0.96–0.29)	<0.001
BAD display			
Front diff.	16.28 ± 12.14 (−4.00–96)	3.59 ± 2.43 (−5.00–8.00)	<0.001
Back diff.	35.74 ± 25.95 (0.00–217)	5.64 ± 3.50 (−3.00–20.00)	<0.001
Dist.Apex-Th	0.84 ± 0.28 (0.13–2.79)	0.82 ± 0.23 (0.18–1.63)	0.349
F.Ele.Th	20.53 ± 14.33 (−7.00–112)	2.29 ± 1.80 (−6.00–8.00)	<0.001
B.Ele.Th	47.39 ± 28.52 (1.00–202)	6.41 ± 3.83 (−2.00–28.00)	<0.001
ProgMin	1.74 ± 1.11 (0.28–11.52)	0.69 ± 0.12 (0.14–1.25)	<0.001
ProgMax	3.20 ± 3.24 (0.91–50.58)	1.22 ± 0.20 (0.75–2.20)	<0.001
ProgAvg	2.26 ± 1.88 (0.74–24.63)	0.96 ± 0.13 (0.65–1.63)	<0.001
ARTmax	187.74 ± 91.00 (1.45–567)	457.83 ± 86.44 (120–725)	<0.001
Df	9.63 ± 8.62 (−1.16–64.58)	0.22 ± 1.14 (−1.85–8.93)	<0.001
Db	8.34 ± 10.57 (−1.00–163.11)	0.04 ± 0.91 (−1.47–3.94)	<0.001
Dp	9.13 ± 12.29 (−1.10–160.47)	0.35 ± 1.89 (−37.00–6.69)	<0.001
Dt	3.02 ± 2.90 (−2.17–32.99)	−0.15 ± 1.02 (–2.68–3.18)	<0.001
Da	2.72 ± 0.81 (−0.72–4.42)	0.27 ± 0.77 (–2.16–2.33)	<0.001
D	8.39 ± 6.22 (0.36–71.96)	0.93 ± 0.66 (−0.79–3.85)	<0.001

**Table 2:** ROC curve analysis for the keratoconus eyes versus normal eyes.

Parameters	AUC	SE	95% CI	*P*	Cutoff	Sensitivity	Specificity
Topographic map							
Kflat (Ant.)	0.755	0.014	0.728–0.783	<0.001	45.15	0.500	0.915
Ksteep (Ant.)	0.857	0.011	0.836–0.878	<0.001	46.45	0.685	0.929
Kmean (Ant.)	0.820	0.012	0.796–0.844	<0.001	45.25	0.646	0.863
Kmax							
Astigmatism (Ant.)	0.815	0.013	0.791–0.840	<0.001	1.65	0.733	0.816
Asphericity (Ant.)	0.795	0.014	0.768–0.721	<0.001	−0.565	0.974	0.618
Kflat (Post.)	0.757	0.014	0.729–0.785	<0.001	−6.55	0.939	0.516
Ksteep (Post.)	0.842	0.012	0.819–0.865	<0.001	−6.85	0.941	0.674
Kmean (Post.)	0.819	0.012	0.795–8.44	<0.001	−6.65	0.945	0.631
Kmax	0.928	0.008	0.914–0.943	<0.001	47.05	0.839	0.935
Astigmatism (Post.)	0.802	0.013	0.776–0.828	<0.001	0.45	0.708	0.828
Asphericity (Post.)	0.780	0.014	0.752–0.807	<0.001	−0.555	0.892	0.678
TCT	0.915	0.08	0.899–0.932	<0.001	506.5	0.890	0.832
Cornea Vol	0.713	0.015	0.701–0.760	<0.001	58.55	0.695	0.687
AC Vol	0.650	0.017	0.617–0.683	<0.001	169.5	0.769	0.452
ACD	0.724	0.015	0.695–0.754	<0.001	3.155	0.703	0.631
AC Angle	0.584	0.017	0.550–0.618	<0.001	33.25	0.854	0.278
Topometric map							
ISV	0.954	0.006	0.942–0.966	<0.001	31.5	0.878	0.962
IVA	0.963	0.006	0.952–0.974	<0.001	0.255	0.913	0.964
KI	0.970	0.005	0.960–0.979	<0.001	1.055	0.910	0.982
CKI	0.824	0.013	0.798–0.849	<0.001	1.015	0.727	0.982
IHA	0.883	0.10	0.863–0.902	<0.001	8.65	0.757	0.886
IHD	0.951	0.006	0.938–0.963	<0.001	0.0175	0.900	0.890
Rmin	0.929	0.008	0.914–0.943	<0.001	7.085	0.968	0.807
HOR Q	0.811	0.13	0.895–0.837	<0.001	−0.495	0.896	0.711
VERT Q	0.719	0.015	0.689–0.750	<0.001	−0.625	0.951	0.545
BAD maps							
Front diff.	0.910	0.008	0.894–0.926	<0.001	8.5	0.716	1
Back diff.	0.954	0.006	0.942–0.966	<0.001	12.5	0.873	0.961
Dist.Apex-Th	0.512	0.17	0.478–0.545	0.499	0.955	0.314	0.760
F.Ele.Th	0.959	0.006	0.947–0971	<0.001	5.5	0.913	0.974
B.Ele.Th	0.967	0.005	0.956–0.977	<0.001	13.5	0.932	0.949
ProgMin	0.935	0.008	0.921–0.950	<0.001	0.925	0.854	0.972
ProgMax	0.964	0.005	0.953–0.974	<0.001	1.675	0.888	0.978
ProgAvg	0.955	0.006	0.943–0.968	<0.001	1.185	0.914	0.951
ARTmax	0.961	0.006	0.949–0.972	<0.001	311	0.966	0.907
Df	0.949	0.006	0.937–0.962	<0.001	2.575	0.847	0.972
Db	0.957	0.006	0.946–0.968	<0.001	1.72	0.882	0.943
Dp	0.954	0.006	0.942–0.967	<0.001	1.855	0.919	0.951
Dt	0.914	0.008	0.897–0.930	<0.001	0.955	0.830	0.888
Da	0.964	0.006	0.953–0.975	<0.001	1.62	0.905	0.968
D	0.972	0.005	0.963–0.982	<0.001	2.615	0.932	0.990

AUC: Area Under ROC Curve.

SE: Standard error.

CI: Confidence Interval.

*P*: Probability.

**Table 3:** Abnormal topometric indices values of the manufacturer's user manual and the study.

Index	Abnormal values of manufacturer's user manual	Abnormal values of the study
ISV		>31.5
IVA	0.28	>0.255
KI	>1.07	>1.055
CKI	1.03	>1.015
IHA	19	>8.65
IHD	0.014	>0.0175
Rmin	<6.71	<7.085

## Abbreviations

K: Keratometry
TCT: Thinnest corneal thickness
Cornea Vol: Cornea volume
AC Vol: Anterior chamber volume
ACD: Anterior chamber depth
AC Angle: Anterior chamber angle
ISV: Index of surface variance
IVA: Index of vertical asymmetry
KI: Keratoconus index
CKI: Center keratoconus index
IHA: Index of height asymmetry
IHD: Index of height decentration
*R*_min⁡_: Minimum sagittal curvature
HOR Q: Horizontal mean asphericity
VERT Q: Vertical mean asphericity
FDE: Subtraction enhanced FBFS from FBFSc
BDE: Subtraction enhanced BBFS from BBFS
Dist.Apex-Th: Distance from corneal apex to thinnest location
F.Ele.Th: Front elevation at thinnest location
B.Ele.Th: Back elevation at thinnest location
ProgMin/Max/Avg: Progress index
ARTmax: Ambrósio Relational Thickness
Df: Deviation of front elevation difference map
Db: Deviation of back elevation difference map
Dp: Deviation of average pachymetric progression
Dt: Deviation of minimum thickness
Da: Deviation of ARTmax
D: Total deviation value.
